# 

**Published:** 1908-08

**Authors:** 


### CONTENTS—AUGUST, 1908.—VOL. XXIV, NO. 2.

DEPARTMENT OF PUBLIC HEALTH—

Report of the Corpus Christi Meeting of Association of Texas Health Officers ........ 47

ORIGINAL ARTICLES—

Retrodisplacement of the Uterus. By Harry K. Leake, M. D., Dallas, Texas ........ 57

Why I Write for Independent Journals. By G. Frank Lydston, Chicago, Ill ........ 66

EDITORIALETS ........ 77

CORRESPONDENCE ........ 78

ABSTRACTS AND SELECTIONS ........ 83

PUBLISHER'S NOTES ........ 84

